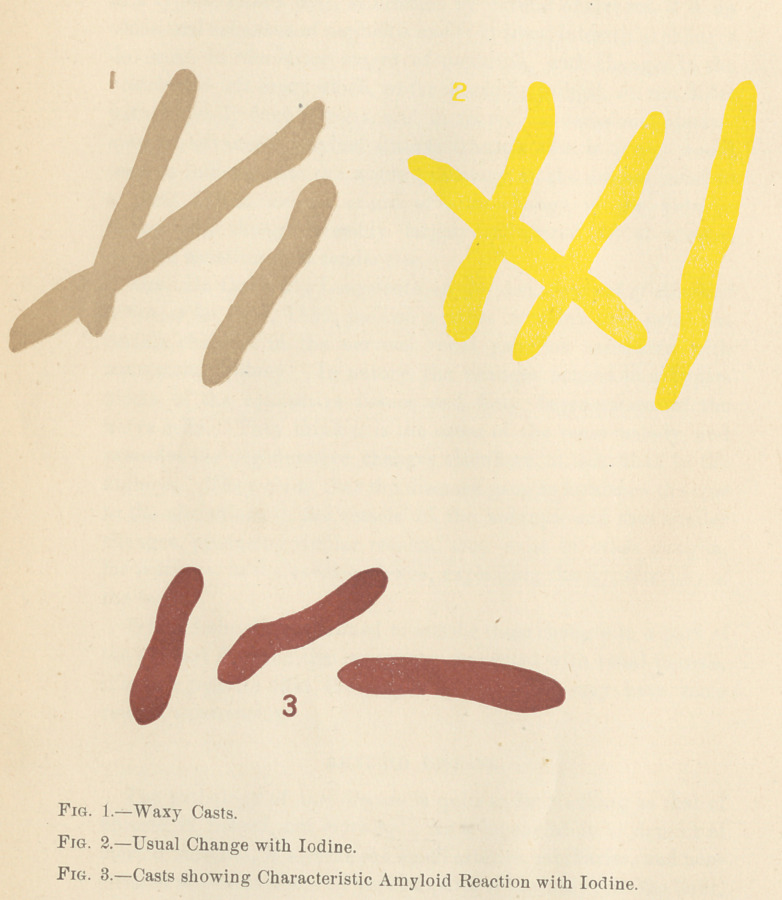# Pathology of Bright’s Disease

**Published:** 1883-04

**Authors:** C. W. Purdy


### ARTICLE II.

### PATHOLOGY OF BRIGHT'S DISEASE. By C. W. PURDY, M.D.

The morbid anatomy accompanying the various lesions and stages of albuminuria has received valuable elucidation from various sources within the past few years. The coarser anatomical textural changes are now pretty well understood, but the more minute microscopical changes which occur in the course of these morbid processes, may be said in some instances still to be obscure. This in itself does not seem so surprising if we consider that the histology of the kidney is not as yet an entirely solved problem.

The nomenclature of the subject also, as at present accepted, is liable to lead to some confusion as to the nature of some of these lesions. Interstitial nephritis for example, would imply an inflammatory process, but it is extremely probable if not actually demonstrated, that the changes occurring in this lesion are in no sense of an inflammatory character.

The pathology of renal diseases is by no means limited to the kindeys themselves, but, in almost every instance, more or less grave departures from normal texture is found to accompany the disease in quite a number of organs remote from the renal. A very comprehensive study of renal pathology would extend far beyond the necessary limits of such a paper as this, and hence we must be content with a mere glance at the more important lesions outside the kidneys and limit the greater portion of what we have to say to the pathology of the kidneys themselves. In studying the history of each special lesion we have seen that symptoms vary very materially with the stage of the disease. It is equally so with the pathology. In the early stages of the lesion we find the slighter forms of textural change, which, followed onward, become more grave and have also added to them others of more or less importance. Hence the pathological history of a lesion becomes a subject very extensive in itself to follow minutely through all its variations. A minute description of all these would furnish sufficient material for quite a hand-book, and hence we shall be obliged to confine ourselves for the most part to a description of well marked typical cases of each lesion, pointing out only the more important variations therefrom which are most commonly encountered.

Following the same order of description dwelt upon in a former paper on "Diagnosis," the first subject to be considered is

### THE PATHOLOGY OF PARENCHYMATOUS NEPHRITIS.

At once the most frequent and the most curable of the forms of Bright's disease, its pathology is probably at present most thoroughly understood. Most authors have divided the disease into several clinical and pathological stages. Grainger Stewart claims for it three distinct stages and describes at length the pathology of each.

The division into acute and chronic stages, however, seems to be the one most commonly followed, and probably corresponds best with the clinical and pathological history, for the changes in all the stages are essentially the same in character, varying only in extent with that of the disease and the length of time they may have been in progress.

If the kidneys be examined after death which resulted during well-marked acute parenchymatous nephritis, the organs will be found considerably enlarged, varying somewhat according to the intensity of the inflammation. The capsule is unaltered, save that it may be congested, and it is stretched. It usually strips off easily. The kidneys are softer and less elastic than in health, and are more easily broken down. The surface is smooth and mottled, and more or less congested.

On making a section of the kidney, it will be at once observed that the cortical portion is much increased relatively. The malpighian bodies are enlarged two or three times their natural size, and stand out prominently as dark red spots. Small extravasations of blood may be seen studded through the cortical, which latter itself is seen to be more or less congested.

The pyramids are deeply congested, exhibiting a deep, dark red color. In cases of extreme swelling of the cortical, their interpyramidal layers crowd in upon the pyramids, compressing their middle portions, and thus forming the wheat-sheaf appearance first pointed out by Rayer. The vascular spaces at the inner edges of the cortical are intensely congested, as are all the smaller renal vessels. In cases of extreme congestion, as where death has resulted from uræmia after sudden suppression of urine, the cortical may assume a darker, dirty brown color.

The microscopical changes are limited to the cortical portion of the organ, for the most part, so far as we have any knowledge. The earliest changes noted are those of a cloudy swelling of the epithelium of the tubes and that of the glomerule. This consists of an infiltration of albuminous granules into the cells, which latter become swollen, more granular and dense, and the cell wall and nucleus less distinct. Occasionally they may contain a few fat drops, though this is more common in the advanced stage. Owing to the enlargement of cells, the tubes become broader in their transverse diameters, and at the same time the lumen of the tube is reduced in size, and sometimes completely closed. As the disease progresses, the tubes become filled with epithelium, free granular matter, and sometimes red blood corpuscles. In some cases, the tubes are filled with a transparent homogeneous material. These becoming detached, carry away with them on their surface a coating of epithelium, and, appearing in the urine, may be seen as epithelial casts. Minute extravasations of blood occupy the tubes in places, which come from the capillaries of the glomerule.

Oertel says that in renal disease following diphtheria, he has found "great numbers of micrococci and exuberant proliferations of the same." Heller claims that he has frequently found in the blood-vessels, in acutely inflamed and swollen kidneys from pyæmia, plugs consisting of spherical bacteria and bacteria-emboli.

In many places the epithelial lining of the tubes desquamates, throwing off entirely the lining. At some places we find the tubes filled quite uniformly with dark masses, products of epithelial growths, blood dèbris or transparent fibrine. In other places these obstructions are only partial, some tubes only being plugged, and this is usually when the disease is of short standing.

As the disease advances and the acute stage to some extent subsides, the epithelium lining the tubes takes on a fatty metamorphosis. The tubes are observed under a high power to be filled with fatty granules mostly contained, however, within the envelope of the epithelial cells. These latter are imbedded in fibrine, which chokes up the tubes in irregular portions. The above changes never occur in the outset of the disease but must be the result of at least three to four months progress.

But, to return to the acute stage of nephritis ; the malpighian bodies stand out prominent, enlarged, and congested, deeply reddened in color. The alterations in the glomerule and its capsule has been the subject of much discussion. Klebs claims that the cavity inside the capsule is filled with angular small nuclei, imbedded in a fine granular mass which almost completely covers the vascular tuft. He claims that this is not not the endothelial lining of the capsule, for, on careful dissection, this endothelium is found very slightly altered, occasionally fattily degenerated. He further claims that the compression of the vascular tufts by this hyperplasia, causes the sometimes sudden diminution or almost entire suppression of the urine and acute dropsy followed by uremia and death.

Johnson, in his lectures on Bright's disease (1874) observes, "That the nuclei in the walls of the capillaries of the malpighian body are abnormally conspicuous." Cornil and Ranvier speak of "a swelling and granular condition of the capsular epithelium, and nuclear proliferation and fatty degeneration of the capillary walls."

Langhans has recently furnished what is probably the most complete and exhaustive description of the changes occurring in the malpighian bodies published to date. He claims three sets of cells are contained within the malpighian body, namely, those lining the capsule of the body; those covering the glomerule or capillary coil; and those of the capillary vessels. All three of these, he claims, share in the pathology of nephritis.

He says, "The epithelium of the capsule in the slight degree of alteration, merely results in overgrowth of cells. The nucleus is larger, more oval, and almost fills the cell. Increase in the number of cells is a more rare occurrence. In the spaces between the layers of cells are found smaller and larger lymphoid cells and more frequent still is partial thickening of the capsular epithelium."

"As to the alterations in the glomerule epithelium, the cells are less adherent; at least, they detach easier as a whole, instead of in part, owing to the dissolving of their connecting plasma. The cells themselves are said to be little altered, sometimes swollen, and when so, the thicker nucleated portion of the cell is the exclusive seat of swelling, making a club-like form of the cell, which may, indeed, separate completely, leaving the free, un-nucleated portion as an independent cell.

"As to the changes in the capillary nuclei, the glomerule is enlarged, and fills closely the capsule. The epithelium of the glomerule, as a rule, is simply swollen, especially its nuclear portion. The lumen of the capillaries is found more or less occupied by a cloudy, more or less granular substance, sometimes containing fat drops. In it are found numerous small, round nuclei These lie in the fine granular mass, and do not adhere to the capillary wall. These capillaries are still pervious to blood under high pressure."

Langhans has never found this condition absent in the large white kidney. Bartels agrees with Langhans that the nuclear proliferation of the capillaries has the effect of diminishing the secretion of urine.

Such, in the main, are the alterations found in the malpighian epithelium in nephritis, as observed by Langhans, consisting, for the most part, first, in cloudy swelling, passing into fatty degeneration in the last stages of the disease, and also into disintegration and exfoliation.

FIG. 1.

FIG. 2.

FIG. 3.

FIG. 1.—Proliferation and thickening of capsular epithelium with compression of glomerule.

FIG. 2.—Desquamative glomerulo-nephritis; surface of the capillaries.

FIG.—Capillary loops with proliferation of the nuclei.

—*After Langhans.*

The intertubular fibrous tissue is subject to slight changes in the later stages of nephritis. In acute cases, as a rule, there are no interstitial changes. When occurring, as they do in the advanced stages, they are doubtless due to long-continued hyperæmia of the kidneys, resulting in hypernucleated overgrowth of the corpuscles of connective tissue. This, with saturations of exudations, and to some extent, also, effusion of white blood cells, increases the spaces between the tubules. The next step in the process is contraction at the expense of surrounding tissue, and hence, in the very advanced stages, we get more or less secondary contraction, according to the degree of interstitial involvement.

This brings us closely in relation to primary granular atrophy, or fibrosis, from which, however, it is not difficult to distinguish by the following characteristics:

In fibrosis, as a rule, the tubes will be found uniformly little, if at all, affected; while in nephritis they will be found in all stages of the changes just pointed out.

In atrophy from fibrosis, the fibrous stroma is very greatly increased; in the inflammatory form it is not increased to any extent. The capsule, too, is more adherent in true fibrosis, and cysts are much more common than in nephritis.

In the acute stage, the organ is everywhere engorged with blood, and congestion is prominent. The cones are darkened; the cortical itself appears a dirty reddish shade, and little spots of extravasated blood may be seen dotted here and there through recent sections, which drip with dark-colored blood. The malpighian bodies stand out enlarged, red and prominent.

After a variable time of perhaps two to four months, if the disease does not terminate in resolution, the chronic stage is entered upon, or, in some instances, the action is less acute from the beginning. The deep congestion has subsided, and the cortical is pale in color. The kidney is large and swollen. The most marked changes now found are the advanced epithelium metamorphoses in the malpighian bodies, and in the tubules already described. In some instances, the interstitial changes are superadded to the above.

The condition of the urine in nephritis deserves special consideration from a chemical standpoint. As a rule, all the natural constituents of the urine are more or less diminished. Albumen is always present, and in proportion to the extent of the disease and in direct ratio to its intensity, usually ranging from 60 to 90 per cent by bulk in well developed cases.

Casts of fibrine containing epithelial cells, or the latter caked together into rods, as molds of the tubules, are found most frequently in the early stages. Granular casts, which are merely epithelium broken down, are found usually somewhat later. Transparent casts of fibrine (small if the tubes retain still their epithelium, large if the tubes have lost their epithelium) are found and known as waxy casts. These are usually found in very advanced stages of the disease.

As to the natural elements of the urine, urea is diminished and directly in proportion to the intensity of the disease or the amount of urine passed. In ordinary cases it is reduced to about one-half, say 14 grammes in twenty-four hours. Extreme diminution is a very ominous symptom and most liable to be followed by convulsions. In convalescence, the urine becoming established again, the urea may rise to 50 grammes.

The diminution of uric acid is less marked and probably does not fall far short of that in health, or it may slightly exceed the latter in some instances.

Phosphates fall about one-third in quantity, which is considerable less of a reduction than takes place in either of the other forms of renal disease. Sulphuric acid is diminished one-sixth to a third, and this reduction is pretty uniform.

Chlorine, which in normal urine is present to the extent of about 8 grammes or a little more, is sometimes totally absent in nephritis. It always suffers greater reduction in this than any other form of Bright's disease. Potash, soda, lime and magnesia are diminished, but we have no reliable estimates as to the exact amounts.

The blood crystalloids are frequently present in the urine in very early stages, often before that of albumen. Dickinson says: "A state of urine in which the crystalloids of the blood only are discoverable is a pre-albuminuric stage, and it would seem that at least in some cases the sapphire blue imparted to the urine by its admixture in a test tube with tincture of guaicum and ozonic ether, declares the presence of those elements of the blood before the less fluent albumen has been able to traverse the coats of the vessels." This is hence a most valuable and early diagnostic of renal disease which is well worth remembering.

RENAL FIBROSIS.

If the kidneys be examined after death which occurred during well developed fibrosis, the organs will be found much smaller than in health, weighing from one to three and a half ounces. They are not always symmetrically reduced in size, one frequently being found considerably smaller than the other.

The surface is found irregularly nodulated and furrowed. The capsule is much thickened and considerably adherent, and consequently is with difficulty stripped off, carrying with it usually detached portions of the corticle. On removal of the capsule, the surface of the organ is seen to be uneven and granular. These granular spots are little hemispherical elevations ranging in size from one-twenty-fifth to one-fifth of an inch, and consist of small knuckles of tubules twisted and contorted from their natural course. These granules are of lighter color than the depressed portions intervening, which latter are slightly vascular. Numerous cysts are usually found scattered over the surface of the kidney varying considerably in size. The consistence of the organ is very much altered, feeling to the finger firmer and less elastic than normal, and on section with a knife it is found tough and resisting. This is more especially the case in the cortical.

On section through the organ it is observed that the cortical is most decidedly altered, being contracted and shriveled up in some cases perhaps to one-sixth of an inch in width. The cones are less affected though a little diminished also, and small cysts are found both in the cortical and medullary portions. The malpighian bodies are much reduced in size, so much so as to be scarcely visible to the naked eye. The small arteries everywhere are much thickened in their coats, and very prominent. Small needles of urate of soda are frequently seen streaked between the intertubular portions of the cones and also scattered throughout the cortical. This is particularly observable in gouty subjects. Da Costa has observed the constant presence of increased amount of fat surrounding the fibrotic kidney, even in emaciated subjects. (See *Am. Jour. Med. Sci.*, vol. lxxx, 1880). The pelvis of the kidney may appear contracted if the cones are little affected, or it may, more often perhaps, be dilated if there is contraction of the cones

Under the microscope the most marked change is found to be an exuberant growth of connective tissue, most observable in the cortex, and destruction of the tubules and small blood vessels. This overgrowth of connective tissue is found in all stages of new development, and is not, be it remembered, a shrinkage of the old connective tissue of which the cortex is sparingly supplied. Dickinson has traced capillaries of evident new growth into this new fibrous tissue in places where it is found in quantity. The most plentiful amount of new fibrous tissue takes its rise at the surface of the cortical, particularly at the depressions between the granular elevations, and extending inwards in rays, envelop thickly the malpighian bodies, the tubules, and small blood vessels.*

The secondary process which invariably attends the growth of new fibrous tissue in other organs, follows the same course here, namely, contraction. The effect of this is to compress, through their thickened capsules the malpighian bodies. The effect on the minute vascular system is to contort, in some places to dilate, but ultimately to contract and obliterate the lumen of many of the capillaries. The tubules are some of them dilated, but more of them contracted, particularly in sections, but for the most part, their epithelial linings are not essentially altered. Dickinson says that any alteration fonnd in the epithelium is merely that of form, the cells appearing more cramped and pressed into angular shapes. The tubes are often occupied by transparent fibrous matter, oil globules are sometimes found through this, or again, the former may crumble into granular material and become packed with casts of this broken up fibrine. The cysts previously spoken of result from contraction or blocking up of the tubes at certain portions, and dilatation of their distal calibers.

On examination under high power, we find the muscular coat of the vessels increased. This thickening of the muscular coat in time is followed or accompanied by nucleolar shrinking, which gradually gives way to coarse transverse fibrillation. Evidences of fatty degeneration follow, particularly at the inner margins of the muscular coat. The outer or fibrous covering of the vessels becomes markedly thickened; under the microscope it appears as if swollen with nearly clear exudative matter (a condition of evident atheroma). This is usually secondary to the changes noted in the muscular coat of the vessels.

This muscular thickening of the vessel coats, advancing to degenerative changes and thickening of the fibrous sheath, it is important to note, is not confined to the vessels of the kidneys, but a similar simultaneous change pervades the systemic arterial tubework, of which we shall have more to say presently. Many of the capillaries in the cortical, in consequence of this thickened over fibrillation of the coat and interspaces, become obliterated, and this is especially noted in the capillary coils of the glomerule.

As to the changes found in the urine, nearly all the natural constituents are diminished except water, which latter is rather increased, except it be in the very late stages.

The urea is always reduced; most so, however, in the advanced stages, averaging from ten to twenty grammes a day in well-marked cases. Uric acid is slightly reduced in the early stages, and in extreme cases it may be totally absent. Phosphoric acid is much diminished, usually to one-fourth the normal quantity; in the late stages there may be but one-fiftieth, or even less. While the amount of phosphoric acid is thus usually much diminished, it varies much, and the quantity is very irregular relatively.

The sulphuric acid is reduced comparatively little to that of the phosphoric, hence it often predominates over the latter.

Chlorine does not suffer much reduction, nor does the chloride of sodium, save in the later stages, when the reduction is very decided.

The alkalies and earths are generally reduced, though the exact extent is unknown.

Of the changes which occur elsewhere in fibrosis, thickening of the cranial vertex may be noted, and this, too, is usually accompanied by thickening of the dura mater, which is adherent to the internal table of the skull. Apoplectic effusions of blood are frequently found in the brain, and sometimes into its ventricles.

I have mentioned elsewhere that visual disorders are a very common complication of renal fibrosis. In the most serious form of this there is swelling of the surrounding disk of the retina, and hypertrophy of the nerve fibers. Virchow likened them to sclerosed ganglion cells. Most authors class them as varicose hypertrophies of nerve fibers. Fatty deposits are found in the fiber or muscular layers of the nerve tissue. The connective tissue fibers of Müller become the seat of fatty changes, giving rise to white streaks on the macular region. The blood-vessels of the retina are subject to the same changes as that in the vessels elsewhere, and hæmorrhages are very constantly to be observed between the retinal layers, or sometimes even in the vitreous humor. The changes described above are those due to what is termed albuminuric retinitis, and, though recurring sometimes in the course of nephritis, are much more commonly the result of granular atrophy. The transient disturbance of vision known as uremic amaurosis is quite another disorder; rapid in its development, it may pass off without leaving any pathological or physiological evidences of its invasion.

The last and most interesting of the pathological changes associated with renal fibrosis, coming under our notice, is hypertrophy of the left ventricle of the heart.

This change, while undoubtedly found in a few cases of nephritis and amyloid disease, especially in their later stages, yet it is doubtful if it occurs sufficiently often in any uncomplicated renal disease save in fibrosis, to entitle it to rank with prominence as a part of the pathology. The fact that hypertrophy of the left heart occurs only in advanced stages of nephritis and amyloid disease, and that it is a constant and early accompaniment of fibrosis is sufficient to relegate it to the pathology of the latter disease. Its occurrence with fibrosis is estimated in frequency by various authors from 40 to 100 per cent. If we seek to unravel the etiological phenomenon of the morbid change, we are forced to admit in the end, that there remains as yet some underlying cause which has not yet been satisfactorily demonstrated.

A very eminent and numerous school claim that as the kidneys fail to eliminate all the waste products of tissue metamorphosis there remains in consequence in the circulation a large amount of foreign substances more or less poisonous, and this renders the blood more resisting to the small vessels and to the tissues, and in order to overcome this resistance the left heart becomes more powerfully developed.

But if we glance at the chemical changes which occur in the urine as the result of nephritis, and compare them with those resulting from fibrosis, we find very little essential difference in the two conditions. Why, then, if this poison theory (if I may so term it) be true, is it that we do not have hypertrophy in nephritis as commonly and as early as in atrophy? for remember, these urine changes are more early by far in nephritis than in atrophy (see table of urine). Again, it would scarcely be in keeping with what we should expect physiologically; if elements which are constantly in the circulation during years of most perfect health, without exerting any morbid change in the heart or vessels; to find that by a moderate increase of these elements in the circulation, so serious an organic change would at once fall upon the heart and vessels; more especially as we look to the circulation as the great prime depurating channel of the system; as the only means of ridding it of the accumulation of these same waste products. We have elsewhere given in full our reasons for disbelieving that any mechanical obstruction in the kidney circulation, due to renal disease, can be the cause of hypertrophy of the heart, and we believe that any such position cannot be maintained. There seems to me much reason in the old and perhaps largely rejected theory of Gull and Sutton, which is that a general fibrotic change, simultaneous with that in the kidneys, occurs in the vessels everywhere, destroying their elasticity, and thus hindering the flow of blood through them to the extent that the heart must increase its power to sustain the circulation. Recent physiologists claim that the blood-vessels do not contract in a vermicular wave from the heart onward, and thus assist the onward current from the ventricle. However that may be, we know that at least, when the fibrotic and muscular change attacks the vessels, they lose their elasticity, and thus resist the cardiac impulse, as we shall attempt to demonstrate. To illustrate this, let us suppose that a heart contracts with a given power, and all the vessels leading therefrom are highly elastic, which we know to be the case in health. With each contracting stroke of the heart these tubes expand quickly, limited only by the force of the ventricular stroke. This stroke of the ventricle being quick and sharply defined, the larger vessels hold for an instant an increased amount of blood. The aortic valves closing sharply at the termination of the contraction of the ventricle, the heart is relieved for a moment of the blood pressure from the vessels. Not so with the aorta and great vessels, which for a moment are dilated to an elastic force equal to the power of the ventricle at the close of its stroke. The vessels filled with blood now exert their elastic force upon it, lessening gradually till they reach their undistended calibers. This elastic vessel force, so long as the aortic valves remain closed, is one entirely independent of the heart. Thus we see, the vessels in a state of health do propel the blood independent of the wave or vermicular theory.

Let us now suppose that the vessels lose their elasticity through degenerative muscular change and fibrotic disease. Let it be supposed that their elasticity is largely abolished, and that they approach in this respect unyielding leathern tubes.

The left ventricle contracting with usual force, the blood responds to the pressure of the stroke, and rushes into the large arterial trunks, and thence through the smaller branches. Instead of meeting everywhere with elastic yielding walls which recede from the force of the blood wave from the ventricle, it everywhere meets with rigid resisting force of unyielding tubes. An extra force therefore, exactly equal to the elastic power of the normal vessels, acts counter to the blood current in the arteries and thus extra resistance must fall largely against the ventricle at the moment of its contraction. The first effect of this is to produce a more prolonged contraction of the left ventricle, because a short quick stroke will not relieve the ventricle of all its contained blood as the inelastic arteries do not receive it so quickly. We hence notice a prolonged systolic sound, heard loudest over the region of the left ventricle.

It requires little reflection on the above facts to show that the effects of diseased vessels must produce increased muscular development of the left ventricle, for increased functional necessity is always met with increased physiological development.

Such have been my own reasonings as to the causes of hypertrophy of the left ventricle of the heart which so constantly accompanies granular disease of the kidney, and to my mind it best explains the philosophy of this interesting morbid phenomenon, as well as its invariable appearance in the early course of of the disease.

But it is asked if hypertrophy only results when and as a result of arterial degeneration, why is it that we find it often recorded as an accompaniment of nephritis and amyloid diseases of the kidneys as well? My own opinion is very strong that it is almost never found to accompany a case of pure uncomplicated nephritis, and the constantly decreasing number of cases now recorded since more advanced and accurate clinical observation has been compared with post mortem observation lends strength to the conviction. Bartels says, "I have never yet succeeded in tracing anatomically in the dead body the transition from inflammatory swelling of the kidney into that of true cirrhosis."

With reference to amyloid disease, however, it must not be forgotten that degenerative changes in the vessels is one of the constant pathological accompaniments of that disease which we shall speak of at length in the proper place. It would not, therefore, surprise us so much to find hypertrophy accompanying amyloid disease as that of nephritis, though this too is rare, for reasons which we shall afterwards explain.

It is proper that we should next consider the nature of the morbid anatomy of renal fibrosis. The name perhaps most commonly assigned to it (interstitial nephritis) would rank it among the inflammations, but it lacks many of the elements of a true inflammation. Seldom preceded by congestion, it on the contrary, takes its origin in the most imperceptible manner, and its march is so extremely tardy that we can scarcely conceive the process to resemble even the more chronic grades of inflammatory processes. Grainger Stewart is perhaps the most emphatic opponent of the inflammatory origin and nature of the disease. He denies that free exudation is to be found in the early stages among the elements of the stroma, and he further points out that excessive formation of connective tissue is frequently found, due to other sources than that of inflammation.

Dr. Handfield Jones advocated ably the non-inflammatory doctrine, and after pointing out that fibrous deposits occur in serous membranes, in certain forms of cirrhotic liver, in the mucous tissues of the stomach, in the testicles, uterus and lungs, he says

"that in all these instances, the process may be from the first non-inflammatory, depending on the exudation of blastema tending abnormally to fibrine development, and not simply maintaining the nutrition of the part." Dickinson, after observing that the disease has nothing in it of inflammatory haste, says: "If the gradual changes in the fibrous tissue which constitute the disorder can be described as inflammatory, the inflammation is of such a slowly progressive sort that it is only in exceptional cases that it is possible to fix the commencement. In the overwhelming majority of instances, the origin of the complaint must be sought, not in chance exposure, nor transient circumstances of any kind, but in influences of a continuous nature." Bartels says with emphasis, "this process leads from its commencement steadily to the dwindling of the substance of the gland, a wasting preceded by *no anterior inflammatory swelling of the gland*." In view of all the facts, the true pathology of fibrosis is probably not of an inflammatory character. The process begins in isolated spots in the cortical, and spreads slowly. If inflammatory in nature, we would expect it to be more diffuse. The most vascular portions of the gland are not the ones the disease is most prone to attack. If free exudation were poured out into a gland the nature of the kidneys, it would affect primarily or simultaneously the tubes, and moreover, we would have as a result free albuminuria, which is not the case.

Lastly, the same process takes place in the fibrous tissue of the vessels early in the disease, pointing to some obscure general systemic influence, whose course it is difficult to reconcile with inflammation in its nature.

Some consider the nature of the changes which cocur in granular atrophy to be a part of the general fibrotic tendency of old age, and the fact that the great majority of cases occur during advancing age would seem to lend some force to this supposition. At the same time, it must not be overlooked that in a few cases the disease has been found in its typical form in children, and here the same simultaneous early changes are found, both in the hypertrophied left ventricle and in the degenerated blood-vessels.

It is more probable, hence, that whatever may be the general systemic influences determining the process, its primary cause

Fig. 1.—Waxy Casts.

Fig. 2.—Usual Change with Iodine.

Fig. 3.—Casts showing Characteristic Amyloid Reaction with Iodine.

and Grainger Stewart is equally emphatic that the process is one of degeneration in existing tissues.

Whatever may be the nature or origin of the morbid process, its recognition is without question a chemical one, resting on its peculiar behavior with iodine solution, which latter instantly turns it a dark brown color wherever found ; be it in the kidneys, liver, spleen, or intestinal vessels.

If the kidneys be examined after death during well-marked amyloid disease, they will be found to present the following characteristics : They are enlarged, perhaps to twice or more than their normal size. As a rule, the capsule strips off easily, showing a pale, smooth, anæmic-looking surface, on which occasionally the stellate veins may be seen prominently.

The organs are usually symmetrically affected. On section, the cortex is found to be increased in thickness, pale, waxy and in thin sections translucent. To the feel it is firmer than normal. The pyramids do not seem essentially altered in color or form. The malpighian bodies are enlarged and are often observed as prominent semitranslucent spots which readily assume a dark brown color when treated with solution of iodine. The capillary walls of the malpighian tufts are thickened owing to infiltration of the peculiar lardaceous matter.

The capillaries of the cortex are similarly involved as are also the vasa recta of the cones.

An exudation of clear glistening material is found in the tubes, the nature of which is not entirely agreed upon. Dickinson thinks it is identical with the amyloid material found in the malpighian bodies and coats of the vessels. Grainger Stewart says it exactly resembles the material of hyaline tube casts, and it does not give the peculiar reaction with iodine. Some observers claim to get the iodine reaction on these casts though not by any means uniformly so, in fact it may be considered an exception rather than the rule.

The tubules themselves are peculiarly affected. Amyloid infiltration without doubt takes place in the basement membrane thereof. The epithelial cells may become enlarged, lose their outlines, and become the seat of fatty deposit. While we thus have swelling both of the epithelium and of the basement membrane and consequent thickening of the tubule, yet, the lumen of the tube does not become contracted or narrowed in its diameter, but, on the contrary, remains remarkably open. This peculiarity becomes very striking on transverse section, when the open end of the tubule preserves its marked rounded outline, like the open end of a gun barrel.

It is not usual for the epithelium of the tubes to desquamate or break down unless some degree of nephritis complicates the case, which it may do in the latter stages, and then, as we would expect, the urine decreases in quantity and we get epithelial casts in the urine. The swelling of the organs is due to thickening of the vessel walls, swollen cells and basement membrane of the tubules, and more or less dilating of the tubes with material forming casts, and swelling of the malpighian bodies from similar causes.

Such are the typical changes found in the amyloid kidney after death, in well marked cases. But the pathology of lardaceous disease is not confined to kidney structures, and moreover, does not originate there in all probability and hence, a brief consideration of the morbid anatomy found in other organs becomes necessary to understand fully the pathology of the disease.

The liver is pretty constantly found enlarged, pale waxy looking, and shows similar translucency at the borders of sections made with the knife. The cells are found enlarged with obscured nuclei. The smaller arteries manifest the same degeneration as those found in the kidneys. These morbid deposits, if we may so term them, respond to the iodine test the same as those in the kidney.

The spleen is almost constantly enlarged in amyloid disease and often enormously so. Here, too, the small arteries are the primary seat of the morbid change which, however, also involves the malpighian bodies and often the pulp.

In the intestines the small arteries become the primary seat of disease, the epithelium of the mucous membrane also degenerates, and the muscular coat of the villi are a prominent seat of the disease. According to Grainger Stewart, however, the substance of the tissues of the villi never becomes affected and rarely the muscular substance of the middle coat. Dickinson points out that, in the intestines the exudation instead of being retained in the tissue, passes off like a secretion from the surface, giving rise to vomiting or diarrhœa, or both.

In the pancreas and lymphatic glands the small arteries are the principal seat of morbid change; rarely if ever does the secreting structure of these glands become involved.

Such are the most common structures involved in the course of amyloid disease. Less often, however, other structures suffer, and indeed, if the records are searched, it would be difficult to find tissues supplied by vessels which are exempt. The muscular fibers of the uterus and the arteries of the vagina are sometimes said to become diseased. Dr. Bennett claims to have discovered characteristic amyloid change in the placenta. In the heart Stewart has distinctly seen waxy vessels. Dr. Gardiner has found the same in cancer, and some observers speak of its presence in the lungs and brain.

As to the chemical alterations found in the urine during the progress of amyloid disease, water alone is increased and very largely so, in all cases till the latter stages, when it becomes diminished, probably as a result of disease of the tubes setting in. Urea is decreased, but not to the extent it is in either of the other forms of renal disease. The average may be placed at 15 to 20 grammes per day. The uric acid varies much. It may even be normal but usually it is diminished, and it is said sometimes to be absent.

Phosphoric acid is always reduced and more uniformly so than in either of the other forms of kidney lesion. It is usually reduced to about one-sixth the normal amount say one-half gramme daily or less.

The sulphuric acid is less marked in its reduction than that of phosphoric and hence it is usually equal to or in excess of the latter averaging about a gramme; chlorine is reduced less than in nephritis but more than in fibrosis.

Alkali salts are below the normal quantity as in other forms of renal disease probably the potash and soda salts are much more so.

Albumen is present, ranging from 40 to 60 per cent. by bulk.

Let us now ask ourselves what is the true pathology of the disease under consideration, and what is the nature of the morbid process resulting in this deposit found so profusely distributed over the vascular system and tissues of so many organs?

Dickinson is a strong advocate of the exudation doctrine and for a most elaborate discussion of this question with many interesting experiments thereon I would refer you to his recent excellent publication on the subject.

It has been shown by analysis that the morbid material is nitrogenous in its composition, and it is probably more closely related to fibrine than any other nitrogenous principle.

In a paper contributed to the fifteenth volume of the Medico-Chirurgical Transactions in 1867, by Dickinson, he thus discusses this question. "Lardaceous material is soluble in alkalies, and the morbid material has been dissolved out by alkalies, leaving the normal tissues almost untouched. If tissue containing the morbid product be washed with water, and afterward with dilute caustic potash, the tissue no longer yields the characteristic amyloid reaction with iodine." That there is a deficiency of potash in the diseased formation, he reasons as follows: "The amyloid reaction is always associated with the condition of acidity, for tissues soaked in a solution of hydrochloric acid will give a deeper tint with iodine than those which are not thus treated. Again, albumen precipitated by an acid will take and retain the color of iodine, which it will not do if not thus acidified. Fibrine, treated with the same acid, gives a striking counterfeit of amyloid material, which in all respects reacts with iodine so nearly similar to amyloid matter that it seems akin to the morbid product. Lastly, the realkalization of the fibrine will render it again insusceptible of lardaceous reaction." He further shows, by statistics of his own and those connected with St. George's Hospital, that four-fifths of the cases of amyloid disease arise from suppurative causes. It is well known that suppuration draws largely on the alkalies of the blood, as proven by the decided alkaline reaction of pus, and also the marked diminution of alkalies in the blood during exhaustive suppuration. The position of Dickinson in this matter at first sight would seem a very reasonable, if not a strong one.

But Ebstein, in a very careful review of the subject, distinguishes between albuminoids proper and the amyloid material very distinctly, as follows: "Their similarity is that they both are closely allied in their chemical composition, both give the xanthoprotein reaction, and respond to Mellon's test, and if boiled in a dilute acid, they conform to leucin and tyrosin in the products into which they subside. But, on the contrary, amyloid matter has greater power of resistance to many solvents, especially its utter insolubility in dilute gastric juice. Again, amyloid material does not putrefy, even in months, and it resists most obstinately suppurative action."

Friedrich affirms that the morbid product results from the gradual transformation of fibrine.

Whatever may be the exact nature of the morbid material which everywhere responds to the iodine solution so characteristically, its method of production is explained in two ways. Dickinson, as before mentioned, claims that it is a free exudation from the blood into the tissues, and in this he is supported by Budd, Portal, Rindfleisch and others.

Grainger Stewart opposes this doctrine with considerable force. He thinks if the process were simply an infiltration, it would not confine itself to the coats of arteries, but would infiltrate rather the softer tissues of organs, and not confine itself to limited portions of the same. He claims that the process is essentially one of degeneration, presenting exactly the characters of such, from the slightest alterations down to disorganization of structures.

I am inclined to believe that the morbid material is the product of albuminoids, modified in some form, first by a predisposition of the general system through certain diseases, such as syphilis, scrofula, suppuration and like processes, which profoundly modify the healthy character of the blood, and that the elaboration or transformation of this albuminoid matter is accomplished at the locations where it is found, for if the change occurred in the blood, then the morbid material should be susceptible of demonstration in that fluid, which, as yet, it never has been.

The process is, therefore, at least very closely allied to degeneration, which, in turn, may be the determination of a succeeding infiltration of the material, already considered.

APPROXIMATE TABLE OF URINE.

| URINE IN HEALTH FOR 24 HOURS. | | NEPHRITIS 24 HOURS. | FIBROSIS 24 HOURS. | AMYLOID KIDNEYS 24 HOURS. |
|---|---|---|---|---|
| Sp. Gr. | 1020 | 1020 to 1040 | 1008 to 1018 | 1002 to 1015 |
| Urea | 512 Grns | 140—250 Grns | 160 to 280 Grns | 256—350 Grns |
| Uric acid | 8½ " | 6—10 " | none—8 " | none—8½ " |
| Phosphoric acid | 48 " | 20—30 " | 2 or 3—24 " | 6—12 " |
| Sulphuric acid | 31 " | 20—25 " | 25—30 " | 22—28 " |
| Chlorine | 1.6 " | none—50 " | 100—120 " | 40—90 " |
| Chloride Sod | 210 " | Diminished | 20—200 " | Greatly reduced |
| Soda | 171 " | " | Diminished | " " |
| Potash | 26—107 " | " | " | " " |
| Water | 35—60 oz | 4—30 oz | Normal or over | Greatly increased |
| Albumen. | | 60 to 90 % by bulk. | 6 to 20 %. | 40 to 60 %. |
| Tube Casts. | | Epithelial (characteristic) hyaline and dark granular casts; blood casts fatty in last stages. | Hyaline, pale granular. In latter stages dark granular, fatty casts. | Not numerous. Hyaline; fatty; waxy; sometimes reacting with iodine. |

## Figures and Tables

**Fig. 1. Fig. 2. Fig. 3. f1:**
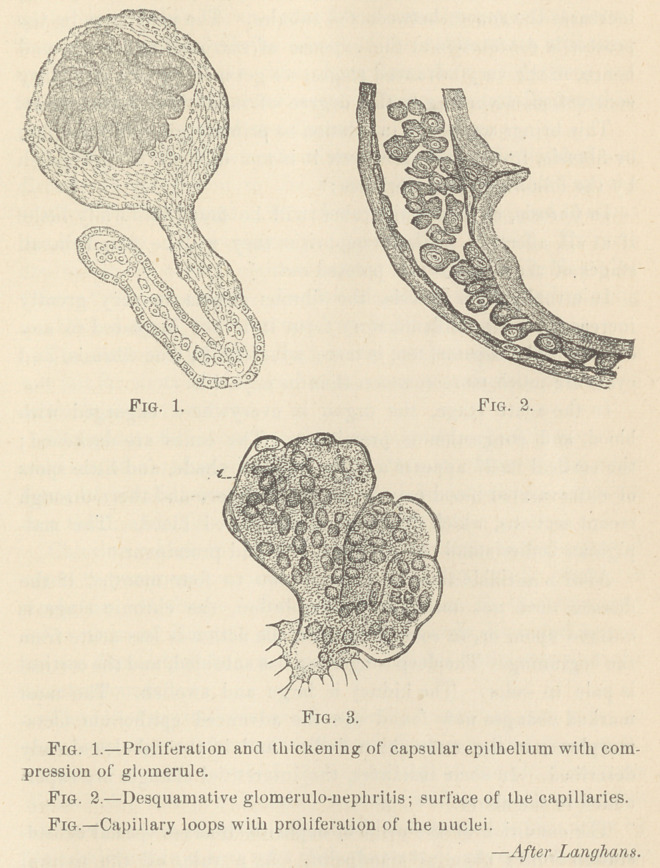


**Fig. 1. Fig. 2. Fig. 3. f2:**